# Online Inspection of Browning in Yali Pears Using Visible-Near Infrared Spectroscopy and Interpretable Spectrogram-Based CNN Modeling

**DOI:** 10.3390/bios13020203

**Published:** 2023-01-29

**Authors:** Yong Hao, Xiyan Li, Chengxiang Zhang, Zuxiang Lei

**Affiliations:** 1 School of Mechatronics and Vehicle Engineering, East China Jiaotong University, Nanchang 330013, China; 2 Key Laboratory of Conveyance Equipment of the Ministry of Education, Nanchang 330013, China; 3 School of Civil Engineering and Architecture, East China Jiaotong University, Nanchang 330013, China

**Keywords:** visible-near infrared spectroscopy, deep learning, online analysis, browned Yali pears

## Abstract

Browning is the most common physiological disease of Yali pears during storage. At the initial stage, browning only occurs in the tissues near the fruit core and cannot be detected from the appearance. The disease, if not identified and removed in time, will seriously undermine the quality and sale of the whole batch of fruit. Therefore, there is an urgent need to explore a method for early diagnosis of the browning in Yali pears. In order to realize the dynamic and online real-time detection of the browning in Yali pears, this paper conducted online discriminant analysis on healthy Yali pears and those with different degrees of browning using visible-near infrared (Vis-NIR) spectroscopy. The experimental results show that the prediction accuracy of the original spectrum combined with a 1D-CNN deep learning model reached 100% for the test sets of browned pears and healthy pears. Features extracted by the 1D-CNN method were converted into images by Gramian angular field (GAF) for PCA visual analysis, showing that deep learning had good performance in extracting features. In conclusion, Vis-NIR spectroscopy combined with the 1D-CNN discriminant model can realize online detection of browning in Yali pears.

## 1. Introduction

Yali pears (*Pyrus bretschneideri* Rehd.) are also known as white pears [1]. With a regular and duck head-like shape, this kind of pear has thin skin, a small kernel, and juicy flesh. Yali pears are loved by consumers at home and abroad because they have the functions of relieving cough and thirst, and clearing heat and detoxification [2]. Yali pears can be easily affected by their environment during storage, especially by carbon dioxide (CO_2_). When the CO_2_ concentration is greater than 1%, the Yali pears will suffer from internal browning [3]. Browning is mainly related to the regulation of various genes and relevant enzymes in Yali pears [4], among which phenolic substances are the main substrates leading to browning. Under aerobic conditions, phenolic substances react with polyphenol oxidase to produce quinones [5], thereby accelerating the occurrence of browning. The browning of Yali pears starts from the inside of the fruit, so it is difficult to observe from the surface at the initial stage. Only when the browning worsens, it can be observed that the surface of Yali pears will become darker. Browning undermines the quality of Yali pears and makes them less tasty, thus leading to bad sales. Therefore, it is urgent to explore a nondestructive, environment-friendly, and accurate technology to identify browning in Yali pears.

During post-harvest storage, Yali pears are prone to core and pulp browning and lose their commercial value. Therefore, huge economic losses are caused to producers and operators. The traditional method of browning discrimination is usually combined with experience to conduct destructive sampling inspection of internal components [6]. This will make fruit analysis difficult to achieve quickly, accurately, and non-destructively. It is not suitable for the actual situation of fruit production and sales at present. Visible-near infrared (Vis-NIR) spectroscopy is an indirect measurement method suitable for rapid online analysis. With its environment-friendly, non-destructive, and flexible integrated detection unit, Vis-NIR spectroscopy has been widely used in the detection of fruit components and defects. Sun et al. [7] adopted Vis-NIR spectroscopy to simultaneously measure online the browned core and soluble solids content (SSC) in Yali pears. They selected a total of 200 samples, including 73 pears with black-heart disease and 127 pears of good quality. The results showed that the classification accuracy of black-heart Yali pears by Vis-NIR spectroscopy was up to 98.3%. The percentages of SSC predictive precision were 97.8% and 99% within deviations of ±0.5 and ±1%, respectively. Qin et al. [6] conducted online and non-destructive detection of moldy-heart disease in apples through the miniature Vis-NIR spectrometer. After the 96 samples were optimized in terms of placement posture, the accuracy of partial least squares discriminant analysis (PLS-DA) reached 93.75%. By adopting Vis-NIR spectroscopy, Hao et al. [8] established an AdaBoost integrated model based on k nearest neighbors (kNN), naive Bayes classifier (NBC), and support vector machine (SVM) for 285 Yali samples. The results showed that the AdaBoost model combined with wavelet transform (WT) had the highest discrimination accuracy (92.63%) of black-heart Yali online detection. Cruz et al. [9] simulated the randomness of fruit positions during spectral acquisition by randomly sampling on the four sides of 1002 ‘Rocha’ pears, so as to figure out the optimal combination of pretreatment method and discrimination method. The results showed that PLS-DA combined with the original spectrum of Yali pears browning classification mode presented the best performance, and the classification accuracy reached 83%. The above results indicated that the research on the discriminant model of browning of Yali pears has basically stayed on machine learning in recent years. These methods are applicable when the amounts of samples are relatively small, and the accuracy requirement is not high. There were few studies on the discriminant of Yali pears by the deep learning model combined with the spectral analysis method. Vis-NIR as an environment-friendly and nondestructive detection technique can be employed to identify browning in Yali pears. When the number of samples is limited, the discrimination accuracy will vary with different identification methods.

In recent years, convolutional neural network (CNN) has been widely and successfully used in image recognition, natural language, video, and other fields. By combining NIR technology with one-dimensional convolutional neural network (1D-CNN), Li et al. [10] developed a data-driven model to estimate the content of organic matter in *Huangshan Maofeng* tea. The results showed that the key features of NIR spectroscopy were successfully extracted, thus providing a new and effective NIR analysis strategy for food analysis. Wu et al. [11] established a quantitative analysis model to detect olive oil content in a corn oil–olive oil mixture by combining Raman spectroscopy with 1D-CNN. The results showed that the 1D-CNN model based on 315 extended average Raman spectra could quantitatively detect the content of olive oil, in which the predictive determination coefficient ($R_{P}^{2}$) and the root mean square error of prediction (RMSEP) were 0.9908 and 0.7183, respectively. Chen et al. [12] established a CNN calibration model for the NIR quantitative determination of water pollution and verified the applicability of shallow convolutional network modeling architecture in feature extraction of one-dimensional spectral data. Rong et al. [13] proposed a detection method based on the principle of deep learning. Through the construction of a 1D-CNN model, the Vis-NIR spectral database containing a total of 500 samples of five kinds of peaches was established for identifying peach varieties. The results showed that the accuracy of the deep learning model reached 100% in the validation data set and 94.4% in the test data set. As suggested by multiple studies, when the number of samples for analysis met certain requirements, CNN combined with Vis-NIR can be applied for the identification of material variety and component analysis. In addition, for the NIR spectral analysis based on scientific research instruments or at a static state, the CNN method can obtain better analytical accuracy, because the spectral response has better wavelength accuracy and less external noise interference.

Vis-NIR online analysis technology cannot be successfully applied without a stable, accurate, and concise modeling algorithm. The effectiveness of the algorithm not only depends on the information extraction ability of the algorithm itself, but also the pretreatment of spectra and effective variable screening before modeling. However, a large number of discriminative models need to be established for the continuous selection of multiple spectral pretreatment methods and variable screening methods, and it is hard to maintain the consistency of optimal variables obtained by different variable screening methods, which affects the stability of the model. For this reason, in order to ensure the accuracy of the model while reducing the complexity and differences in the selection of pretreatment methods and spectral variable screening methods, this paper intends to adopt a deep learning method to simultaneously realize the automatic extraction of spectral feature variables and model construction. In other words, the depth features of Vis-NIR of Yali pears are extracted and used for modeling by means of a 1D-CNN algorithm, so that the interference of noise on the feature variables selection can be avoided and the stability of the model is thus improved. This study can provide reference for rapid online analysis of Yali pear quality in an accurate and reliable manner based on deep learning.

## 2. Materials and Methods

### 2.1. Sample Preparation

The Yali pear samples in this paper were collected from an orchard in Hebei Province, China, and then were refrigerated and transported to the laboratory immediately after harvesting. In order to reduce the influence of temperature on spectrum acquisition [14] and the damage caused by rapid cooling to the fruit, these samples were stored for 24 h under constant temperature before experiments. The sample storage temperature was 1 °C (±0.5), and the humidity was 85–95%. The diameter of Yali pears selected in the experiment was about 75 mm, and they had no obvious defects and mechanical damage on the surface. They were cleaned and numbered. A total of 495 samples were collected, including 256 healthy pears and 239 browned pears. The Kennard–Stone algorithm was used to divide healthy and browned pear samples into calibration sets and test sets in the ratio of 7:3.

### 2.2. Vis-NIR Spectroscopy Acquisition

**Figure 1:**
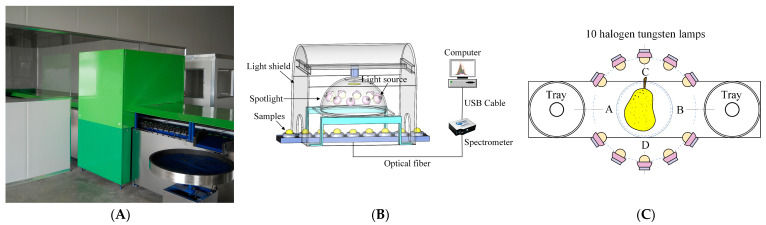
Dynamic online acquisition device of Yali pears: (**A**) device diagram; (**B**) core detection unit; (**C**) schematic diagram of arrangement and distribution of tungsten halogen lamps.

The Vis-NIR spectra of Yali pears were collected by an Ocean Optics INC, QE65Pro (Dunedin, FL, USA) high-precision spectrometer with a wavelength range of 361–1165 nm. The spectral resolution of ca. 0.8 nm and the signal-to-noise ratio (SNR) of 1000 were employed. Before spectral acquisition, the spectrometer was preheated for 30 min to obtain stable light source energy. Then, a PTFE ball was used to conduct the qualified calibration on the spectrometer and the sorting device. Figure 1 shows the dynamic online acquisition device of Yali pears. The online detection device, which consists of spectrometer, light source, conveyor belt, optical fiber (model QP1000-2-VIS-NIR, Ocean Optics, Dunedin, FL, USA), and computer and so on, includes three modules (transmission module, spectral acquisition module, and control module).

The spectrometer integration time was set to 80 ms, and the conveyor belt can transmit six samples per second. The optical fiber probe was located below the tray, at a distance of about 12 mm from the conveyor belt. The light source, which consists of ten 100-watt halogen tungsten lamps (Osram), was located above the sample and arranged along the concentrating coil at an angle of 45 degrees. The halogen tungsten lamps were arranged in an equidistant arrangement of five lamps on both sides of the Yali pear samples, as shown in Figure 1C.

Yali pears were manually placed on the device for spectral acquisition, and the acquisition process is as follows. First, as shown in Figure 1C, the pear samples were placed in a way that the connecting line direction between the pears’ stalk and the pears’ pedicel was always perpendicular to the running direction of the conveyor belt. When the pears were transported to the spectral acquisition station by a conveyor belt, the arc-shaped equidistant light source on both sides could evenly penetrate the pears into the detector probe. Second, the spectrometer was triggered by signals sent by the system encoder, which acquired the Vis-NIR spectrum of Yali pear samples. The collected transmission spectra were automatically stored on the computer. Each sample was scanned five times in order to ensure the consistency of spectral collection, and the average spectrum was used as the final spectrum of the sample. Each sample spectrum had 1044 variables in the wavelength range of 361–1165 nm. As the signal-to-noise ratios at both ends of the detector were low, 1024 variables in the wavelength range of 387–1165 nm were thus reserved for subsequent experiments. The whole spectral acquisition process was completed in the designed shield that effectively avoided the influence of external stray light.

### 2.3. Evaluation of Browning in Yali Pears

**Figure 2:**
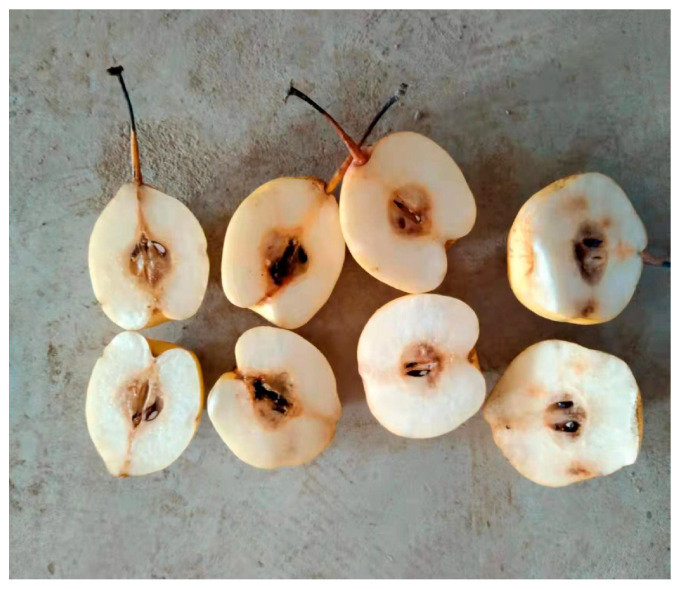
Photo of the internal pulp of pears with different degrees of browning.

The damage information of browning in Yali pears was evaluated by the destructive method. After spectral acquisition, each pear sample was cut along the direction of the CD line in Figure 1C, and three experts with years of experience in pear planting determined whether the inside of these pears had browned. The evaluation criteria are as follows: if there were no abnormal changes of cell tissue near the pears’ core, then it was a healthy pear; while if tissues around the core were browned, then it was classified as a browned pear. The picture of flesh inside browning pears is shown in Figure 2, and it can be clearly seen in the figure that there are varying degrees of browning near the core.

### 2.4. Spectral Pretreatment and Variable Selection

The original Vis-NIR data contain not only feature information, but also many noise and other interference factors, such as the temperature and humidity of the environment during the spectral acquisition process, the location of the samples, and the light source stability. In order to achieve modeling spectra with better quality and enhance the features with stronger correlation in the spectra, the traditional pretreatment methods such as first-derivative (SG 1st−Der) [15], multiplicative scatter correction (MSC), standard normal variable transformation (SNV) [16], moving average smoothing (MAS), and wavelet transform (WT) [17] were used in this experiment for modeling. In this case, problems of baseline shift, scattering, and high-frequency noises of the original spectra can be removed.

The spectra of Yali pear samples contain many uninformative variables which are characterized by a highly overlapping spectral band. In order to eliminate redundant and uninformative spectral variables, Monte Carlo uninformative variable elimination (MCUVE) was used in this paper to select effective wavelengths, which can significantly reduce the number of feature wavelengths and the complexity of the model, as well as improve the generalization ability and robustness of the model [18]. In MCUVE, the contributions of wavelength variables in high dimensional spectral data were evaluated by making full use of the intrinsic correlation among samples. The contribution value of each spectrum, which served as an indicator of stability, was sorted to establish a series of partial least squares regression (PLSR) models. The importance of each variable was measured by its stability value, and the threshold value of variable optimization was finally determined by the minimum root mean square error of cross-validation. 

### 2.5. Construction Method of Discriminant Model

#### 2.5.1. PLS-DA Model

PLS-DA, or partial least squares discriminant analysis, is a discriminant analysis method used to deal with classification and discrimination problems in high-dimensional data analysis. It can effectively distinguish the observed values between classes and find the influencing variables that lead to the differences between classes [19]. In addition, PLS-DA can reduce the influence of multicollinearity among variables. A simple linear model can be used to describe the relationship between variables and responses, as shown in Equation (1).

(1) $$Y=b_{0}+b_{1}X_{1}+b_{2}X_{2}+\ldots +b_{n}X_{n}$$

where *b*_0_ refers to intercept, *b_i_* is the regression coefficient of the sample, and *X_i_* is the *i*th spectral response value corresponding to the wavelength.

#### 2.5.2. SVM Model

The basic principle the support vector machines (SVM) algorithm is to figure out the separation hyperplane that can correctly partition the training data sets and maximize the geometric interval and map the input vectors to a high-dimensional feature space using a specific transfer kernel function [20]. The construction of a linear surface for feature space endows the SVM network with higher generalization ability. The optimization objectives of SVM are presented as follows.

(2) $$\left\{\begin{array}{l} \underset{\omega ,b,\xi_{i}}{\min}\frac{1}{2}{\left\|\omega \right\|}^{2}+C\sum_{i=1}^{n}\xi_{i}\\ s.t.\left\{\begin{array}{l} y_{i}(\omega \cdot x_{i}+b)\geq 1-\xi_{i}\\ \xi_{i}\geq 0,\;i=1,2,\ldots ,\;N \end{array}\right. \end{array}\right.$$

where *ω* is the normal vector that determines the direction of the hyperplane; *b* is the displacement term which determines the distance between the hyperplane and the original point; *ζ_i_* is the relaxation variable which represents the degree to which the sample does not satisfy the constraint; *C* is the penalty factor, and the greater its value is, the larger value indicates a greater penalty for classification; *n* is the number of samples; *x_i_* is the supporting vector of training sample; *y_i_* is the category of the corresponding sample.

#### 2.5.3. 1D-CNN Model

**Figure 3:**
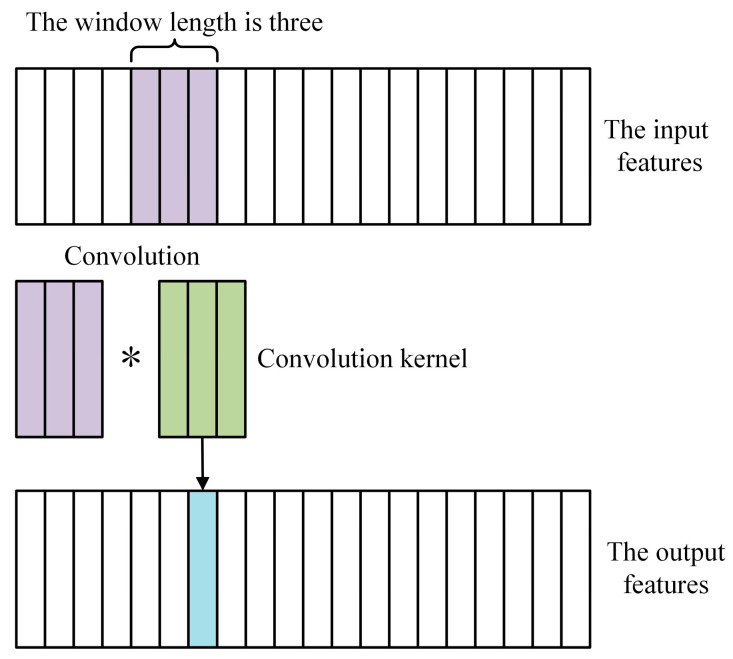
Schematic diagram of 1D-CNN.

Integrating feature learning and classification, the convolutional neural network (CNN) is a feed-forward neural network with convolution computation and deep structure. By mapping simple data features to complex high-dimensional space by convolution and pooling, CNN can obtain the accuracy of classification. Studies have shown that the convolution layer can extract important features with more useful information while removing noises and unimportant features, which is a more robust algorithm for feature extraction than the feature selection method [21]. The working principle of 1D-CNN is shown in Figure 3, where the dimension of the convolution kernel is 3 × 1 and the moving step is 1. The data features are extracted through distributively moving the convolution kernel.

**Figure 4:**
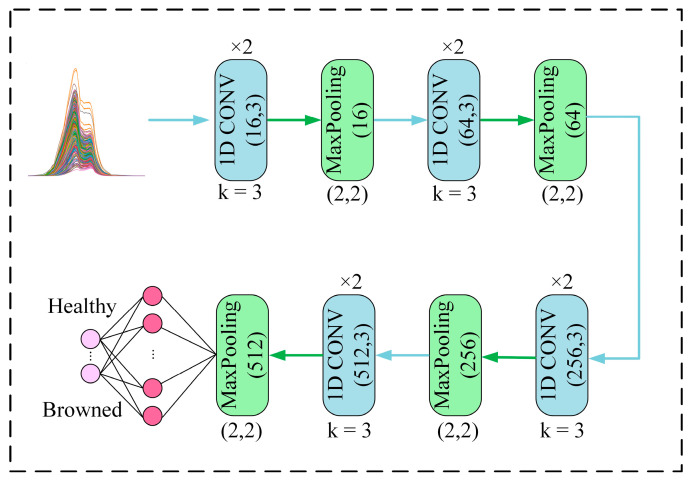
The flow chart of the network framework of the 1D-CNN model.

The structure of the 1D-CNN network consists of the convolution layer, pooling layer, and full connection layer. The operation of the convolution layer is equivalent to the process of pretreatment and feature extraction in traditional machine learning [22]. Before convolution, the size of the convolution kernel and the number of filters need to be set and these two parameters are obtained through multiple experiments. The maximum pooling was selected as the pooling layer, which can reduce the dimension of feature mapping without losing important information, thus cutting computation amount and time of network training. The fully connected layer acted as the classifier in CNN and optimizes the network as the feature combination layer. In order to prevent over-fitting in training data, a dropout layer was added to the network structure to suppress over-fitting by removing some neurons. The flow chart of the network framework of the 1D-CNN model is shown in Figure 4.

All models and chemometric procedures used throughout the work were implemented based on Python 3.8.

### 2.6. Evaluation of Models

Three indexes including the overall accurate identification rate (*Accuracy*), accurate identification rate of healthy pears (*RH*), and accurate identification rate of browned pears (*RB*) were adopted to evaluate the online discriminative model of browning in Yali pears. *Accuracy* refers to the rate of correct identification made by the classifier for all samples; *RH* refers to the percentage of correct identification made by the classifier for all healthy samples; *RB* refers to the percentage of correct identification made by the classifier for all browned samples. The greater the values of these three indexes are, the higher the rate of correct classification is. *Accuracy*, *RH*, and *RB* can be calculated according to the following equations.

(3) $$Accuracy=\left(1-\frac{He+Be}{H+B}\right)\times 100\%$$

(4) $$RH=\left(1-\frac{He}{H}\right)\times 100\%$$

(5) $$RB=\left(1-\frac{Be}{B}\right)\times 100\%$$

where *H* refers to the total number of healthy samples, and *He* refers to the number of healthy samples being mistakenly classified. *B* is the total number of browned samples, and *Be* is the number of browned samples being mistakenly classified.

## 3. Results and Discussion

### 3.1. Vis-NIR Spectral Analysis of Yali Pears

**Figure 5:**
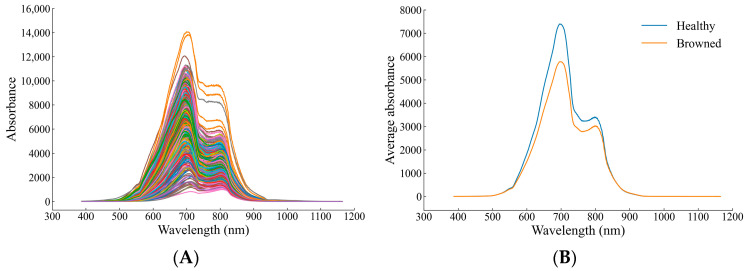
Vis-NIR spectra of healthy and browned pears: (**A**) raw spectra; (**B**) average spectra.

Figure 5 shows the original Vis-NIR spectra of healthy and browning Yali pears, as well as the averaged spectra. It can be seen from Figure 5A that the spectra of healthy pears and browning pears overlap so seriously that it is impossible to directly distinguish from the spectral graph whether Yali pears were browned or not. It can be seen from Figure 5B that the average spectrum of healthy pears is higher than that of browned pears on the whole, and the band of 600–800 nm is the most obvious, which may be due to the strong absorption of transmitted light by browning tissue inside the fruit. As shown in the spectral graph, there are two absorption peaks at approximately 700 and 800 nm. In addition, the absorption peak at around 700 nm may result from the stretching and contraction of the fourth overtone of the C-H functional group, while that at around 800 nm may be related to the stretching and contraction of the third overtone of the N-H functional group [23].

### 3.2. PCA Analysis on Health Status of Yali Pears with Different Pretreatment Methods

In the experiment, principal component analysis (PCA) was applied to analyze the spectral spatial distribution of two kinds of Yali pear samples pretreated by different methods. The core idea of PCA is dimensionality reduction, and its working principle is to transform a group of variables that may have correlation into a set of linearly uncorrelated variables, namely principal components, through orthogonal transformation. The data in the new subspace defined by the principal component are usually easier to interpret [24].

**Figure 6:**
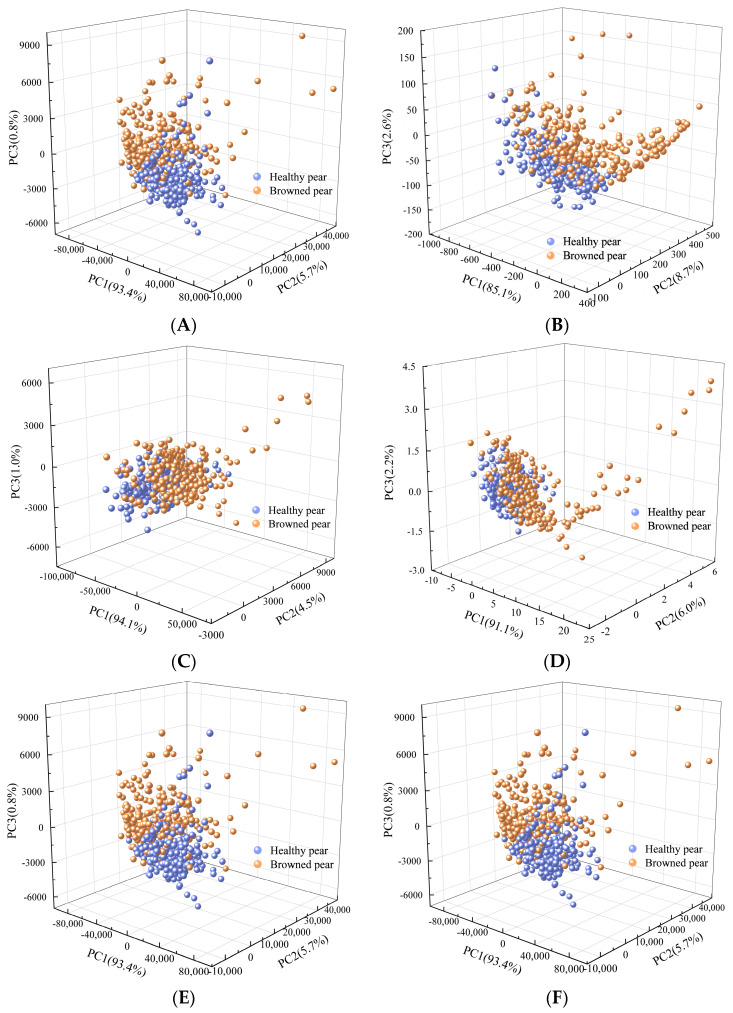
The spatial distributions of the first three principal components of healthy pear and browned pear samples pretreated by different methods: (**A**) raw; (**B**) SG 1st−Der; (**C**) MSC; (**D**) SNV; (**E**) MAS; (**F**) WT.

The data set contains 256 healthy pears and 239 browned pears. PCA analysis was conducted on the original spectrum and on the spectra separately pretreated by SG 1st−Der, MSC, SNV, MAS, and WT. The first three principal components were retained for visualization. The results are shown in Figure 6. It can be observed that the spatial distribution of spectra pretreated by MAS and WT were basically consistent with that of the original spectrum. The spectral points of browned pears and healthy pears were so crossed that they cannot be distinguished, indicating that these two pretreatment methods failed to significantly improve the spectral features of data used in this experiment. Compared with the original spectrum, pretreatment methods of SG 1st−Der, MSC, and SNV have shown better performance. The spectral points of samples pretreated by these three methods presented a trend of classification separation in terms of spatial distribution. The cumulative contribution rates of the first three principal components of SG 1st−Der, MSC, and SNV were 96.4%, 99.6%, and 99.3%, respectively. The cumulative contribution rates of MSC and SNV were both over 99%, indicating that both methods were applicable to the experimental data. In order to further explore the effectiveness of the method, PLS-DA and SVM models were established combined with different pretreated methods. The results of the discrimination are shown in Table 1. It can be seen from the table that the discrimination accuracy of SNV is higher than that of MSC. Therefore, the SNV pretreatment method was finally selected for model optimization in this experiment.

### 3.3. Robust Variable Selection Based on MCUVE Method

**Figure 7:**
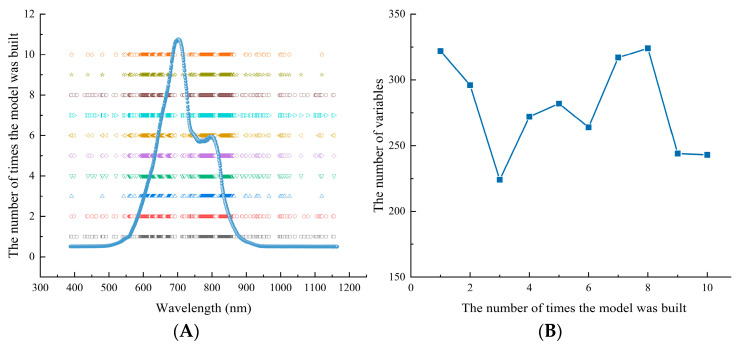
The results of variables selected after 10 runs of MCUVE method: (**A**) variable distribution; (**B**) the number of variables retained.

When the spectrum contains a large number of invalid variables, it will undermine the quality of modeling to a certain extent. In order to further simplify the model and improve the accuracy and interpretability of the model, the spectral wavelength was optimized by the MCUVE method after SNV pretreatment. As variables were selected in a random way, each model was selected 10 times. The optimized results are shown in Figure 7. Figure 7A shows the corresponding relationship between the variable distribution of the 10-time wavelength selections and the corresponding spectrum. It can be seen that the wavelength distribution of each optimal selection is not completely consistent, but most of the variables are concentrated at the absorption peak of the sample. Figure 7B shows the number of variables retained after 10 runs of MCUVE, and it can be seen that the number is within the range of 224 to 324. Although the distribution of the selected variables is relatively concentrated, the number of retained variables is different. When the distribution of the modeling samples is not representative or the number is small, although the MCUVE method can improve the accuracy of the model, the number of wavelengths selected for multiple times is unstable, which affects the robustness of the model.

### 3.4. Evaluation Model for Health Status of Yali Pears Based on PLS-DA Method

PLS, which contains least squares regression analysis and discriminant analysis, is suitable for analyzing cases where the differences between groups are small while differences within groups are large. Unlike PCA, PLS-DA is a supervised discriminant analysis statistical method, which is widely used in the discrimination of Vis-NIR spectral data analysis. The core idea of PLS-DA is to obtain the optimal number of factors (LVs) in the calibration set through Monte Carlo cross-validation combined with an F-test. In this experiment, 10-fold cross-validation was employed to determine LVs. Furthermore, the results show that the root mean square error of the calibration set was the smallest when the LVs were 9, indicating that the model presented the best classification performance when the LVs were 9.

The model was established based on the calibration set, and then its performance was verified by the sample test set. The modeling results of the full spectrum, the pretreated spectrum, and the optimally selected spectrum (the best model in the 10 selections is chosen, whose number of variables is 264) are shown in Table 2. It can be seen that after spectral pretreatment and variable selection, the discrimination accuracy of discriminating quality of Yali pears had been improved. The optimal model of PLS-DA is SNV-MCUVE-PLS-DA, and accuracy, RH, and RB of its test set are 97.32%, 100%, and 92.16%, respectively.

### 3.5. Evaluation Model for Health Status of Yali Pears Based on SVM Method

SVM is a class of generalized linear classifiers that perform binary classification of data based on the supervised learning method. SVM mainly maximizes the interval between different categories by finding the division hyperplane with the largest interval. It is a class of generalized linear classifiers for binary classification of data in a supervised learning manner. The selection of SVM hyperparameters (kernel function, penalty factor *C*, and kernel function parameter *g*) is determined by an optimizing algorithm through grid search so as to obtain the best calibration model. Grid search is an exhaustive search method, which uses cross-validation to optimize the estimation function and finally solves the optimal parameters. In this experiment, grid search determines that linear is the optimal kernel function, the value of the optimal penalty factor *C* is 100, and the value of the optimal kernel function parameter *g* is 0.001.

It can be seen from Table 3 that the optimal SVM model is SNV-MCUVE-SVM, and accuracy, RH, and RB of its test set are 98.66%, 100%, and 96.08%, respectively; moreover, the modeling results of the SNV-SVM model are exactly the same as those of the SNV-MCUVE-SVM model. There are only 317 variables involved in the modeling of the SNV-MCUVE-SVM model, which greatly reduces the complexity of the model. Therefore, the optimal SVM model is the SNV-MCUVE-SVM model in which spectra are pretreated by SNV and variables are optimally selected by MCUVE.

### 3.6. Evaluation Model for Health Status of Yali Pears Based on 1D-CNN Method

The above research shows that although traditional methods can classify the browning pears, the process is cumbersome. A detection model with good performance requires rich theoretical knowledge and practical experience of the modeler. For this reason, it is necessary to figure out the effect of the deep feature extraction method on discriminating browned pears and healthy pears.

This paper proposed building a 1D-CNN model based on one-dimensional Vis-NIR spectral data in a bid to determine more accurately whether Yali pears are browned or not. The network was composed of an input layer, convolutional layer, pooling layer, and full connection layer. These layers were mainly used for feature extraction and classification. Among them, the convolutional layer and the pooling layer were used to extract the features of the data, while the fully connected layer was used to map the previously extracted features to the output space for subsequent classification. The activation functions of the convolutional layer and the full connection layer were the Relu function and the Sigmoid function, respectively. The dropout rate was set to 0.3. The Adam optimizer was adopted to optimize the network, the learning rate of the training model was set to 0.0001, the batch size was set to 64, and the number of iterations (epochs) was set to 500. The output feature value of the full connection layer which can combine features was set to 400.

The spectral data of the calibration set was read into the initialization network for iterative training, while the spectral data of the test set was used to evaluate the accuracy of the deep learning model. The deep learning model was randomly run 10 times in order to test its robustness, with the training samples and test samples unchanged. The modeling results of 10 runs are shown in Table 4. It can be seen that the correct discrimination rate of the 1D-CNN discriminant model has the lowest rate of 96.64% and the highest rate of 100%. The difference between the two is only 3.36%, indicating that the model is relatively robust.

### 3.7. Establishment and Error Analysis of the Optimal Evaluation Model for Health Status of Yali Pears

PLS-DA, SVM, and 1D-CNN discriminant methods were separately used to establish online models for identifying healthy pears and browned pears. These models were then adopted to qualitatively discriminate healthy pears and browned pears that were not involved in the modeling. In order to optimize the model and improve the discriminant performance of the model, the spectral preprocessing and variable selection were performed before PLS-DA and SVM modeling. As errors of spectral rotation and shift were eliminated, the model became less complicated. After the network was completely designed, the 1D-CNN discriminant model can be directly constructed based on the original spectrum of Yali pears, because 1D-CNN had excellent performance in integrating pretreatment and extracting features. 

**Figure 8:**
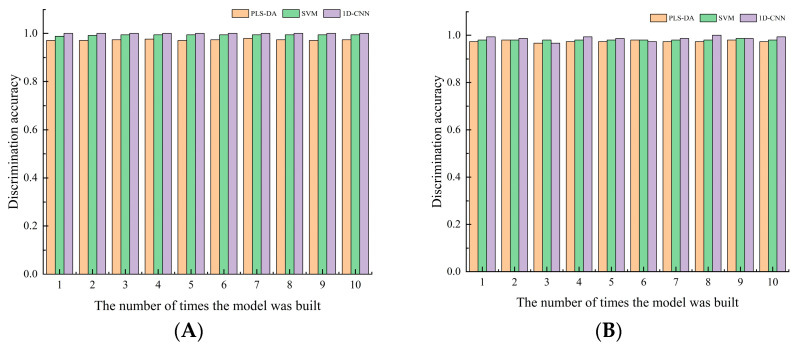
Discrimination accuracy of training sets and test sets in 10 PLS-DA, SVM, and 1D-CNN models: (**A**) training sets; (**B**) test sets.

The discrimination results of training sets and test sets in 10 PLS-DA, SVM, and 1D-CNN models are shown in Figure 8. It can be seen that 1D-CNN has better discriminant performance than PLS-DA and SVM, as evidenced by better modeling and testing accuracy of all its discriminant models. Moreover, in the 1D-CNN method, there is no requirement for preprocessing and variable selection of sample spectra. The model with the highest discrimination accuracy in 10 times of 1D-CNN modeling is selected as the final model, and the discrimination results are shown in Table 5.

It can be seen from the table that the PLS-DA, SVM, and 1D-CNN models all can correctly identify healthy pears, but there are also cases in which browned pears are misjudged as healthy pears. From the perspective of the data in experiment, the overall discrimination performance of the 1D-CNN model is better than that of PLS-DA and SVM models, as the discrimination accuracy of its test set was as high as 100%. Furthermore, the calculation time of the 1D-CNN model in predicting the test set samples was about 0.0256 s, which met the requirements of online classification. Compared with the traditional models, the 1D-CNN model can automatically identify important features and has better classification performance. Furthermore, this model is so simple to operate that even those without much basic knowledge of modeling can use it properly. In summary, the 1D-CNN model is the optimal model for discriminating browned pears.

### 3.8. Deep Feature Analysis on Vis-NIR Spectra of Yali Pears

**Figure 9:**
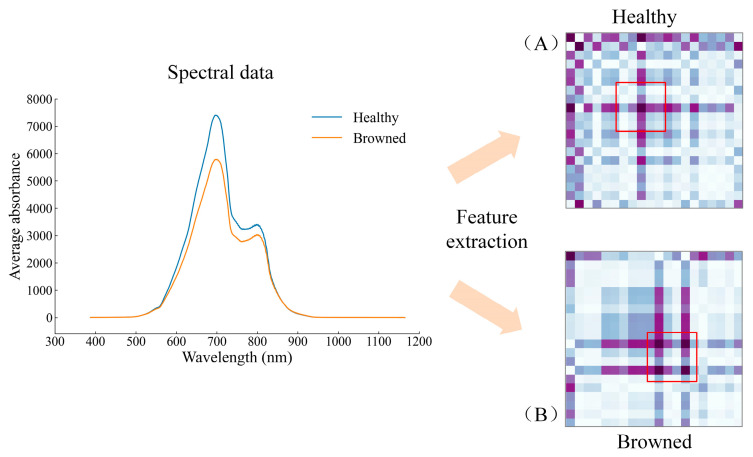
GAF coding process of spectral data of Yali pears.

It can be seen from the above studies that the deep learning model has excellent performance in extracting features of Vis-NIR spectral analysis for Yali pears. In order to carry out a further expression and analysis of the spectral features extracted by the 1D-CNN method, the Gramian angular field (GAF) was used to transform the spectral data into graphs. The GAF has been used for visual expression of one-dimensional time series signals and the good classification results have been obtained [25,26]. The main advantage of the GAF is that features of the original one-dimensional spectral signal of the image during encoding are maintained and bidirectionally mapped to the two-dimensional image [27]. The feature data extracted by 1D-CNN are converted into a 20 × 20 image by the GAF. The GAF encoding process of the Yali pears’ spectral data is shown in Figure 9. Figure 9A,B show the results of the GAF transformation of healthy pears and browned pears, respectively.

**Figure 10:**
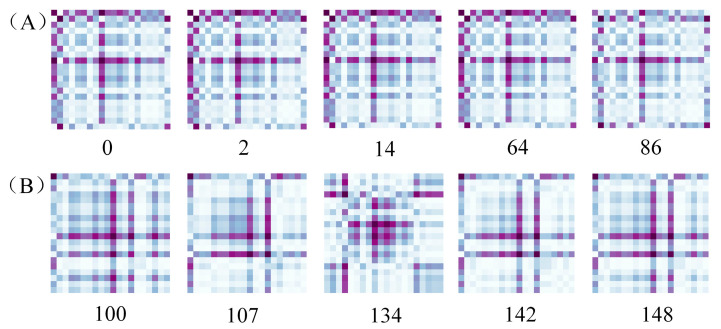
GAF graphs of spectra of some pear samples: (**A**) healthy pears; (**B**) browned pears.

Figure 10 shows five pictures randomly selected from the two types of pear samples. Among them, Figure 10A,B correspond to the GAF expression of healthy and browned pears in the test set, respectively. It can be seen from Figure 10 that the model can adaptively learn the separable features from the original spectral information. The GAF graphs of healthy pears are similar (all in the shape of ‘+’); the GAF graphs of browned pears are slightly different due to the different browning degrees of tissue cells near the fruit core, but most of the graphs are shown in Figure 10B (in the shape of ‘#’). It can be distinguished that the GAF graphs of the two types of samples are quite different, which further confirms that the deep learning model works excellently in feature extraction and classification. Meanwhile, the local and global features of Vis-NIR which can be easily classified and distinguished, if integrated by the feature combination layer (the full connection layer), can save calculation time while retaining important information.

**Figure 11:**
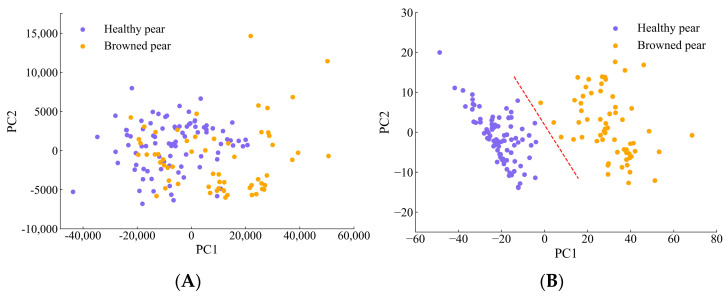
Two−dimensional spatial distribution of pear samples: (**A**) raw spectral data; (**B**) feature data extracted by 1D−CNN method.

The GAF features were visualized by the PCA method. The two-dimensional feature distributions of the original spectral data and of features extracted by the 1D-CNN are shown in Figure 11A,B. It can be seen from Figure 11A that the spatial distribution of the original spectral data is relatively chaotic; the spectral points of the two types of samples overlap each other, so the feature distinction is not obvious. It can be seen from Figure 11B that the data features of 1D-CNN after dimensionality reduction by PCA have better aggregation and feature distinction.

## 4. Conclusions

Based on the in-depth study of 1D-CNN, this paper proposed a device and method for online detection of Yali pear browning using Vis-NIR spectroscopy combined with deep learning. SVM, PLS-DA, and 1D-CNN models were established to discriminate the browned pears. The original spectrum and five spectra pretreated by SVM and PLS-DA methods were used to establish and optimize the discriminative model for browning in Yali pears. Moreover, the MCUVE method was adopted to reduce the complexity of the model. The experimental results show that the energy spectra processed by SNV and MCUVE were adopted to establish the PLS-DA discriminant model, and the discrimination accuracy rate was 97.32%; the energy spectra processed by SNV and MCUVE were used to establish the SVM discriminative model, and the discrimination accuracy rate was 98.66%. Without cumbersome pretreatment and variable screening, the 1D-CNN discriminative model has shown the optimal performance, and the discrimination accuracy rate was as high as 100%. In conclusion, Vis-NIR spectroscopy combined with the 1D-CNN discriminant model can realize online detection of browning in Yali pears.

## Figures and Tables

**Table 1:** The discrimination results of PLS-DA and SVM models’ training sets under different preprocessing methods.

Model	Pretreatment	Accuracy (%)	LVs	Model	Pretreatment	Accuracy (%)
PLS-DA	Raw	82.66	4	SVM	Raw	96.53
	SG 1st−Der	95.09	5		SG 1st−Der	100
	MSC	95.66	8		MSC	100
	SNV	97.11	9		SNV	100
	MAS	84.97	5		MAS	96.25
	WT	85.26	4		WT	95.66

Polynomial order of SG 1st−Der is three, and the number of smoothing points is 15. The number of smoothing points in MAS is 15. Daubechies 8 wavelet is selected in WT.

**Table 2:** The results of PLS-DA discriminative models for pear quality based on different modeling variables.

Model	Method	Accuracy (%)	RH (%)	RB (%)
PLS-DA	Raw	80.54	88.64	68.85
	SNV	95.97	98.98	90.20
	SNV-MCUVE	97.32	100	92.16

RH: accurate identification rate of healthy pears; RB: accurate identification rate of browned pears.

**Table 3:** The results of SVM discriminative models for pear quality based on different modeling variables.

Model	Method	Accuracy (%)	RH (%)	RB (%)
SVM	Raw	95.97	97.73	93.44
	SNV	98.66	100	96.08
	SNV-MCUVE	98.66	100	96.08

**Table 4:** The results of 10 parallel runs of the 1D-CNN discriminant model for pear quality.

Modeling Method	The Model Number	Accuracy (%)	RH (%)	RB (%)
1D-CNN	1	99.33	100	98.36
	2	98.66	98.86	98.36
	3	96.64	98.86	93.44
	4	99.33	100	98.36
	5	98.66	98.86	98.36
	6	97.32	95.45	100
	7	98.66	100	96.72
	8	100	100	100
	9	98.66	98.86	98.36
	10	99.33	100	98.36

**Table 5:** Statistics of test results of SVM, PLS-DA, and 1D-CNN models.

Samples in Test Set	SNV-MCUVE-PLS-DA		SNV-MCUVE-SVM		1DCNN	
	Healthy	Browned	Healthy	Browned	Healthy	Browned
Discrimination	98	51	98	51	88	61
Misclassification	0	4	0	2	0	0
RH (%)	100		100		100	
RB (%)	92.16		96.08		100	
Accuracy (%)	97.32		98.66		100	
Prediction Time (s)	0.0113		0.0099		0.0256	

## Data Availability

The data used to support the study findings are available from the corresponding authors upon request.

## References

[1] Han D., Tu R., Lu C., Liu X., Wen Z. (2006). Nondestructive detection of brown core in the Chinese pear ‘Yali’ by transmission visible–NIR spectroscopy. Food Control..

[2] Guang Y., Li W., Wen S., Chang S. (2021). Research Progress on Functional Ingredients and Food Development of Ya Pear. Farm Prod. Process..

[3] Du Y., Wang W., Jia X., Le W., Wei J., Guan J. (2017). Development Status and Proposal of Pear Storage Industry of Hebei Province. Storage Process.

[4] Wang Z., Zhang Y., Li Y., Li L., You L., Li X., Jin Z., Yan S. (2020). Relationship Between LAC Gene Expression and Core Browning of Yali Pear. Sci. Agric. Sin..

[5] Ren Z., Cheng Y., Guan Y., Zhang Z., Guan J. (2021). Effects of Mechanical Damage on Browning in ʻYaliʼ Pear. Food Science and Technology. Food Sci. Technol..

[6] Qin K., Chen G., Zhang J., Fu X. (2021). Optimization of Fruit Pose and Modeling Method for Online Spectral Detection of Apple Moldy Core. Spectrosc. Spectral Anal..

[7] Sun X., Liu Y., Li Y., Wu M., Zhu D. (2016). Simultaneous measurement of brown core and soluble solids content in pear by on-line visible and near infrared spectroscopy. Postharvest Biol. Technol..

[8] Hao Y., Wang Q., Zhang S. (2021). Study on Online Detection Method of “Yali” Pear Black Heart Disease Based on Vis-Near Infrared Spectroscopy and AdaBoost Integrated Model. Spectrosc. Spectral Anal..

[9] Cruz S., Guerra R., Brazio A., Cavaco A.M., Antunes D., Passos D. (2021). Nondestructive simultaneous prediction of internal browning disorder and quality attributes in ‘Rocha’ pear (*Pyrus communis* L.) using VIS-NIR spectroscopy. Postharvest Biol. Technol..

[10] Li M., Pan T., Bai Y., Chen Q. (2022). Development of a calibration model for near infrared spectroscopy using a convolutional neural network. J. Near Infrared Spectrosc..

[11] Wu X., Gao S., Niu Y., Zhao Z., Ma R., Liu H., Zhang Y. (2022). Quantitative analysis of blended corn-olive oil based on Raman spectroscopy and one-dimensional convolutional neural network. Food Chem..

[12] Chen H., Chen A., Xu L., Xie H., Qiao H., Lin Q., Cai K. (2020). A deep learning CNN architecture applied in smart near-infrared analysis of water pollution for agricultural irrigation resources. Agric. Water Manag..

[13] Rong D., Wang H., Ying Y., Zhang Z., Zhang Y. (2020). Peach variety detection using VIS-NIR spectroscopy and deep learning. Comput. Electron. Agric..

[14] Fan S., Li J., Xia Y., Tian X., Guo Z., Huang W. (2019). Long-term evaluation of soluble solids content of apples with biological variability by using near-infrared spectroscopy and calibration transfer method. Postharvest Biol. Technol..

[15] Chen H., Pan T., Chen J., Lu Q. (2011). Waveband selection for NIR spectroscopy analysis of soil organic matter based on SG smoothing and MWPLS methods. Chemom. Intell. Lab. Syst..

[16] Hao Y., Wu W., Shang Q., Geng P. (2019). Analysis Model of Oleic and Linoleic Acids in Camellia Oil via Near-Infrared Spectroscopy. Acta Optica Sinica..

[17] Diwu P., Bian X., Wang Z., Liu W. (2019). Study on the Selection of Spectral Preprocessing Methods. Spectrosc. Spectral Anal..

[18] Lao C., Chen J., Zhang Z., Chen Y., Ma Y., Chen H., Gu X., Ning J., Jin J., Li X. (2021). Predicting the contents of soil salt and major water-soluble ions with fractional-order derivative spectral indices and variable selection. Comput. Electron. Agric..

[19] Mansuri S.M., Chakraborty S.K., Mahanti N.K., Pandiselvam R. (2022). Effect of germ orientation during Vis-NIR hyperspectral imaging for the detection of fungal contamination in maize kernel using PLS-DA, ANN and 1D-CNN modelling. Food Control..

[20] de Santana F.B., Otani S.K., de Souza A.M., Poppi R.J. (2021). Comparison of PLS and SVM models for soil organic matter and particle size using vis-NIR spectral libraries. Geoderma Reg..

[21] Yan K., Zhou X. (2022). Chiller faults detection and diagnosis with sensor network and adaptive 1D CNN. Digit. Commun. Netw..

[22] Tian S., Wang S., Xu H. (2022). Early detection of freezing damage in oranges by online Vis/NIR transmission coupled with diameter correction method and deep 1D-CNN. Comput. Electron. Agric..

[23] Zou X., Zhao J., Povey M.J.W., Holmes M., Hanpin M. (2010). Variables selection methods in near-infrared spectroscopy. Anal. Chim. Acta..

[24] Jaillais B., Pinto R., Barros A.S., Rutledge D.N. (2005). Outer-product analysis (OPA) using PCA to study the influence of temperature on NIR spectra of water. Vib. Spectrosc..

[25] Jiang J., Guo M., Yang S. (2021). Fault diagnosis of rolling bearings based on GAF and DenseNet. J. Mine Autom..

[26] Yao L., Sun J., Ma C. (2022). Fault Diagnosis Method for Rolling Bearings Based on Grame Angle Fields and CNN-RNN. Bearing.

[27] Liu S., Wang S., Hu C., Bi W. (2022). Determination of alcohols-diesel oil by near infrared spectroscopy based on gramian angular field image coding and deep learning. Fuel.

